# Simple reaction times to cyclopean stimuli reveal that the binocular system is tuned to react faster to near than to far objects

**DOI:** 10.1371/journal.pone.0188895

**Published:** 2018-01-05

**Authors:** Gábor Horváth, Vanda A. Nemes, János Radó, András Czigler, Béla Török, Péter Buzás, Gábor Jandó

**Affiliations:** 1 Institute of Physiology, University of Pécs Medical School, Pécs, Hungary; 2 Department of Ophthalmology, Kantonsspital, St. Gallen, Switzerland; Centre de neuroscience cognitive, FRANCE

## Abstract

Binocular depth perception is an important mechanism to segregate the visual scene for mapping relevant objects in our environment. Convergent evidence from psychophysical and neurophysiological studies have revealed asymmetries between the processing of near (crossed) and far (uncrossed) binocular disparities. The aim of the present study was to test if near or far objects are processed faster and with higher contrast sensitivity in the visual system. We therefore measured the relationship between binocular disparity and simple reaction time (RT) as well as contrast gain based on the contrast-RT function in young healthy adults. RTs were measured to suddenly appearing cyclopean target stimuli, which were checkerboard patterns encoded by depth in dynamic random dot stereograms (DRDS). The DRDS technique allowed us to selectively study the stereoscopic processing system by eliminating all monocular cues. The results showed that disparity and contrast had significant effects on RTs. RTs as a function of disparity followed a U-shaped tuning curve indicating an optimum at around 15 arc min, where RTs were minimal. Surprisingly, the disparity tuning of RT was much less pronounced for far disparities. At the optimal disparity, we measured advantages of about 80 ms and 30 ms for near disparities at low (10%) and high (90%) contrasts, respectively. High contrast always reduced RTs as well as the disparity dependent differences. Furthermore, RT-based contrast gains were higher for near disparities in the range of disparities where RTs were the shortest. These results show that the sensitivity of the human visual system is biased for near versus far disparities and near stimuli can result in faster motor responses, probably because they bear higher biological relevance.

## Introduction

Identification of relevant objects in the visual scene is important for successful interaction with our environment. Stereopsis is a visual function by which the visual system decodes the spatial arrangement of our surroundings [1]. Horizontal disparity (to which we simply refer in the following as disparity) is quintessential in this process and it is the result of the slightly different viewing positions of the two eyes. Among others, disparity and motion are important visual cues helping the segregation of the visual scene to identify favorable and avoidable objects.

One of the pioneers in the field of binocular perception was Béla Julesz [2, 3], who invented static and dynamic random dot stereograms (DRDS) [4] to study binocular mechanisms. In Julesz’s random dot stereograms, parts of the corresponding images are shifted laterally, whereby disparity is introduced to an extent that the visual system is still able to fuse the two images [1]. The degree of the disparity is used as an input to the binocular neuronal networks to compute depth information in the brain [5–7] enabling stereoscopic perception.

An important feature of Julesz’s random dot stimuli is that the sensation of depth or a shape separated from the surface is only visible binocularly provided the viewer has intact stereovision. Such stimuli, which are free from monocular cues and contain disparity information only, are called “cyclopean” after Julesz.

The measurement of simple reaction times (RT) provides information about the speed of early neural processing of visual stimuli [8]. The effects of various properties of visual stimuli, such as luminance, contrast and spatial frequency on simple RTs have been extensively studied [9–15]. Plainis and Murray [16] have found that the relationship between RT and contrast can be described with sufficient accuracy by the modified form of the so-called Piéron function:

$$RT={RT}_{0}+\frac{1}{C}$$ (1)

where, *RT* is the reaction time, *RT*_*0*_ is the asymptote RT, *k* represents the slope of the curve and *C* is the Michelson’s contrast [16]. This relationship allows extracting a measure of contrast gain (essentially 1/k) from a series of RTs at supra-threshold contrast levels.

By using this technique, Plainis and Murray [17] were able to identify two mechanisms, one with low gain and high spatial frequency preference and another with high gain and low spatial frequency preference that they linked to the parvocellular and magnocellular pathways, respectively.

There is evidence for multiple channels in binocular disparity processing [18, 19]. It has long been suspected for example, that fine (up to around 20 minutes of arc) and coarse disparities are detected by different mechanisms [20, 21]. This is also supported by clinical observations that fine and coarse stereopsis can be selectively lost while the other function still remains intact [22].

There is another type of potential dichotomy between near (crossed) and far (uncrossed) disparities. Several studies have demonstrated clear asymmetries between the detection mechanisms of near and far disparities. Woo and Sillanpaa measured smaller absolute stereoscopic thresholds for near than for far disparities. Stereo disparity thresholds [23] as well as diplopia thresholds [24] were reported to be significantly lower for near disparities. Further studies have shown overall lower discrimination errors [25–27], shorter decision reaction times [26] and duration thresholds [28] for near disparities in a near far discrimination task. Asymmetries between the role of near and far disparities in surface construction were also pointed out by Ishigushi and Wolfe [29]. Thus, the bulk of the evidence from psychophysical studies points towards higher sensitivity to near disparities. Furthermore, some authors raised the possibility that the neuronal mechanisms responsible for the processing of near and far disparities are different [29–31]. This may be due to the higher biological relevance of objects near to or moving towards the organism. We hypothesize that near disparity targets should therefore evoke faster motor responses, which may be reflected in faster simple reaction times. Surprisingly, simple RTs to cyclopean stimulus targets have never been studied systematically.

The aim of this study was to measure simple reaction times to targets embedded in dynamic random dot stereograms in order to examine the effect of a range of near and far disparities. The stimuli covered a wide variety of disparities, including the fine (3–20 arc min) and coarse (20–120 arc min) ranges. In addition, we used two contrast levels in order to estimate contrast gain for each disparity and thus reveal the effects of binocular disparity while factoring out the effect of contrast.

## Methods

### Participants

Fifteen young, healthy adults (7 males, 8 females, 14 right and one left handed, between 20–31 years) participated in the study. The observers were naive to the purpose of the experiment and had normal or corrected-to-normal vision (Visual acuity was 5/5 or higher). Intact binocular vision was confirmed with a random dot stereotest [32]. According to the test, all participants recognized the orientation of four Snellen E targets in DRDS at 0.8 arc min disparity with 100% accuracy. All procedures were approved by the Regional and Local Research Ethics Committee of the Clinical Center at the University of Pécs and they were carried out in accordance with the relevant institutional and national regulations and legislation and in accordance with the World Medical Association Helsinki Declaration as revised in October 2008. Participants provided their written consent to participate in this study.

### Apparatus

Stimuli were generated on a high performance personal computer and presented on a polarized flat-panel LCD 3D monitor (LG Cinema D2343P) at 60Hz refresh rate. Observers wore circularly polarizing glasses supplied by the manufacturer throughout the experiments. Participants were seated in a dark room at 1m viewing distance and were asked to fixate on a central mark, a black dot of 36 arc min diameter. The viewing angle was optimized for each participant by presenting a rivaling test pattern (i.e., horizontal stripes for the right and vertical stripes for the left eye) to eliminate cross talk between the left and right channels. The central area 1080 * 1080 pixels (16° * 16° visual angle) was used for stimulus presentation and the rest of the screen remained black.

### Stimulus calibration

The luminance of the monitor (all of the scales from 0 to 255 of the colors red, green, blue and grey levels) was measured using a photometer (ILT-1700 Photometer, International Light Technologies, Peabody, USA) for the left and right channels through the polarizing glasses and without the glasses as well. The aims of the calibration procedure were: 1) to maintain constant mean luminance of 30 cd/m^2^ for all conditions, 2) to provide control over stimulus contrast and 3) avoid interocular differences in luminance or contrast. This was achieved by adjusting the gray levels of the dark and bright dots independently for each channel using our custom made iterative least square algorithm. Details of this procedure have been described by Markó et al. [32] and were adapted to the current display technology. The algorithmically calculated values were tested with a psychophysical method by presenting the participants patterns consisting of binocularly correlated and anti-correlated horizontal or vertical stripes. The gray levels of the dots were then adjusted until the orientation of the stripes could not be detected monocularly.

### Stimuli

Stimuli were dynamic random dot stereograms (DRDSs) generated by the same custom made software as used in our previous study [33]. The images were composed of randomly placed bright and dark dots filling the stimulus area in a 1:1 ratio, each dot subtended 3.7 arc min. Each random dot was updated at 60Hz.

**Fig 1:**
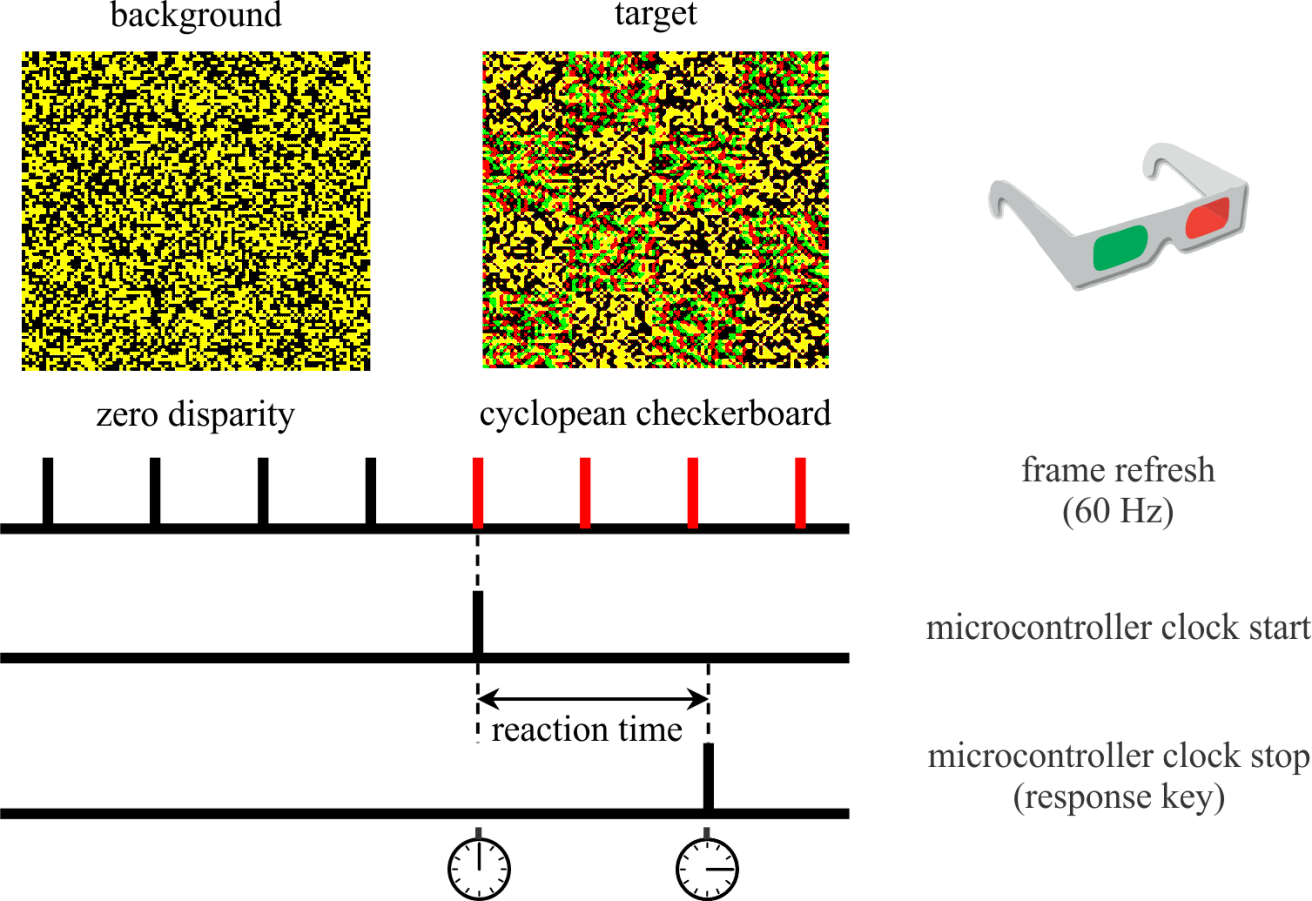
Measurement of reaction times. The random dot stereograms at the top of the figure can be viewed using simple red-green goggles. The cyclopean checkerboard appears in near disparity if the red and green filters are in front of the left and right eyes, respectively. Disparity turns into far if the filters are reversed. Note that this is just an illustration of concept for the reader, in the real experiment, left and right channels were separated by circularly polarizing filters and the pattern of random dots was updated at 60 Hz frequency. The images show one frame each of the background (left) and the target (right) condition. Reaction time was measured with millisecond accuracy from the first frame of the target (red tick marks) until the response button was pressed.

The target stimulus was a checkerboard pattern composed of zero disparity checks and other checks having the actually tested disparity (Fig 1). The checks subtended 120x120 arc min. When the cyclopean stereograms were viewed through polarizing glasses and fused correctly, every other check appeared to emerge from, or to drop behind the fixation plane. When either eye was covered while wearing the polarizing glasses, all stimuli appeared as a random spatio-temporal noise and the target could not be detected. We generated the target patterns by calculating two random dot matrices, one with zero and one with the desired near or far disparity. The checkerboard was then patched together from the two matrices with equal areas taken from the two patterns. This method implies that as disparity increases, increasing margins of the disparate surfaces became binocularly uncorrelated. When disparity reached the size of the checks (i.e. 120 arc min), the target regions became binocularly uncorrelated for both types of disparity.

Since the perception of stereograms containing uncorrelated surfaces can be quite diverse [34] and depend on individual factors, we did not aim to standardize stimulus appearance. Instead, we kept the retinal stimuli identical for near and far disparities of the same magnitude, except that images for the two eyes were swapped.

### Experimental design

Eight disparity values (3.7, 7.3, 11, 15, 18, 29, 58, 120 arc min) and two contrast levels (90% (high-contrast), 10% (low-contrast)) comprised a block of 16 stimulus conditions. Conditions were shuffled within the block. All participants were tested for 10 consecutive repetitions of the entire block resulting in 160 trials in one session. Near and far disparities were tested in different sessions separated by several weeks in order to exclude learning or fatigue. Thus, we obtained 320 reaction times from each participant. We performed a statistical coherence analysis and found that the number of RT measurements per condition and the number of participants were sufficient to achieve reliable statistical outcome. See S1 Appendix for further details.

### Measurement of reaction time

Zero disparity DRDS (i.e., correlated random dots with no visible pattern) with a central fixation mark was presented as a background throughout the experiment except when the target appeared. Each trial started with a static delay of 4s after which the central fixation mark turned white, serving as a cue for the participant to fixate and not to blink. The target appeared with a further delay jittered between 0 and 1s and was presented for a constant duration of 20 frames (333 ms). The observer was asked to press the response button with the thumb of the dominant hand as quickly as possible after detecting a change in the pattern of the stimulus other than the constant updating of the random dots. Since the targets were disparate relative to the background and they were not visible to either eye alone, they could only be detected by the stereo system. Thus, any differences in RT had to be derived from binocular processing and/or the transmission of its signals to motor systems. The checkerboard pattern was visible even if disparity was as high as 120 arc min and thus well beyond Panum’s fusional limit.

The timing and control of the RT measurement is demonstrated in Fig 1. A custom-made microcontroller system (Arduino, Scarmagno, Italy) measured the time with millisecond accuracy from the first frame of the target until the response button was pressed. The precision of this system was verified by an independent measurement using a photodiode and an oscilloscope.

RTs between 140–800 ms were accepted and in case of an invalid RT, the same target was presented once again. If the observer failed on two consecutive runs, the stimulus was categorized as “not seen” and a different stimulus condition followed. The fraction of trials in the pooled data of all participants where the stimulus was not seen was never higher than 10% for any of the stimulus conditions (Tables 1 and 2). In the second step, all values outside ±2.0 standard deviations from the mean of each stimulus condition were excluded from further analysis. The fraction of outliers removed due to this criterion was never higher than 8.6% for any stimulus condition (Tables 1 and 2). According to the Lilliefors test, the RT distribution for some participants and stimulus conditions was significantly different from the normal distribution (p<0.05). This is not surprising, since RT distributions tend to be skewed to the right, therefore the median was used for further statistical analysis.

**Table 1:** Distribution of the number of trials with valid RTs for near disparities.

	90% contrast level								10% contrast level							
**disparity (arc min)**	3.7	7.3	11	15	18	29	58	120	3.7	7.3	11	15	18	29	58	120
**not seen**	0	0	0	0	0	0	0	0	3	1	0	0	0	0	9	10
**outliers**	9	5	5	8	8	5	9	3	4	8	7	7	8	6	3	7
**valid RTs**	141	145	145	142	142	145	141	147	143	141	143	143	142	144	138	133
**total**	**150**	**150**	**150**	**150**	**150**	**150**	**150**	**150**	**150**	**150**	**150**	**150**	**150**	**150**	**150**	**150**

Stimuli “not seen” and outliers within pooled data of the 15 participants. Outliers were defined as those outside the mean ±2 SD. Only the valid RTs were used in the statistical analysis.

**Table 2:** Distribution of the number of trials with valid RTs for far disparities.

	90% contrast level								10% contrast level							
**disparity (arc min)**	3.7	7.3	11	15	18	29	58	120	3.7	7.3	11	15	18	29	58	120
**not seen**	0	0	0	0	0	0	0	0	2	0	0	0	0	1	1	4
**outliers**	6	6	7	3	7	5	8	6	3	2	1	8	4	7	5	2
**valid RTs**	144	144	143	147	143	145	142	144	145	148	149	142	146	142	144	144
**total**	**150**	**150**	**150**	**150**	**150**	**150**	**150**	**150**	**150**	**150**	**150**	**150**	**150**	**150**	**150**	**150**

Stimuli “not seen” and outliers within pooled data of the 15 participants, Outliers were defined as those outside the mean ±2 SD. Only the valid RTs were used in the statistical analysis.

### Data analysis

Statistical analysis was performed using SPSS 20.0 (SPSS Inc., Chicago, USA). Repeated measures ANOVA (rANOVA) was used to analyze the main effect of stimulus disparity and contrast. Reported are *F*-values, *p*-values and effect sizes (*r*). Mauchly’s test indicated that the assumption of sphericity was violated for the main effect of disparity, therefore the degrees of freedom were adjusted using the Greenhouse–Geisser correction when it was necessary. Pairwise comparisons were made following Bonferroni-correction. Homoscedasticity of participant data was confirmed by Levene’s test, thus the participants were pooled for further analysis. Significance level was set at p<0.05. Since calculation of RT-based contrast gain (Eq 2) involves the reciprocal of RTs, gains were log transformed before statistical testing in order to better approximate normal distribution. Gain data outside of the mean ± 3 SD were replaced by the high or low, respectively, of the remaining data set excluding these outliers. The statistical models for each result section is detailed in S1 Table. In addition, we re-analyzed our data using a single 3-factor (type of disparity, contrast, magnitude of disparity) repeated measures ANOVA design. Its results summarized in S8 Table supported our main conclusions.

## Results

The general aim of the present study was to explore the effect of a range of near and far binocular disparities on simple reaction times and on contrast gains calculated on the basis of the contrast dependence of reaction time.

Overall, the statistical analysis indicated that the magnitude of stimulus disparity had significant effect on reaction times. In general, we found a characteristic U-shaped tuning as a function of disparity. These data sets are shown in Figs 2–5 and S1–S7 Tables. Data will be analyzed in detail in the following sections.

**Fig 2:**
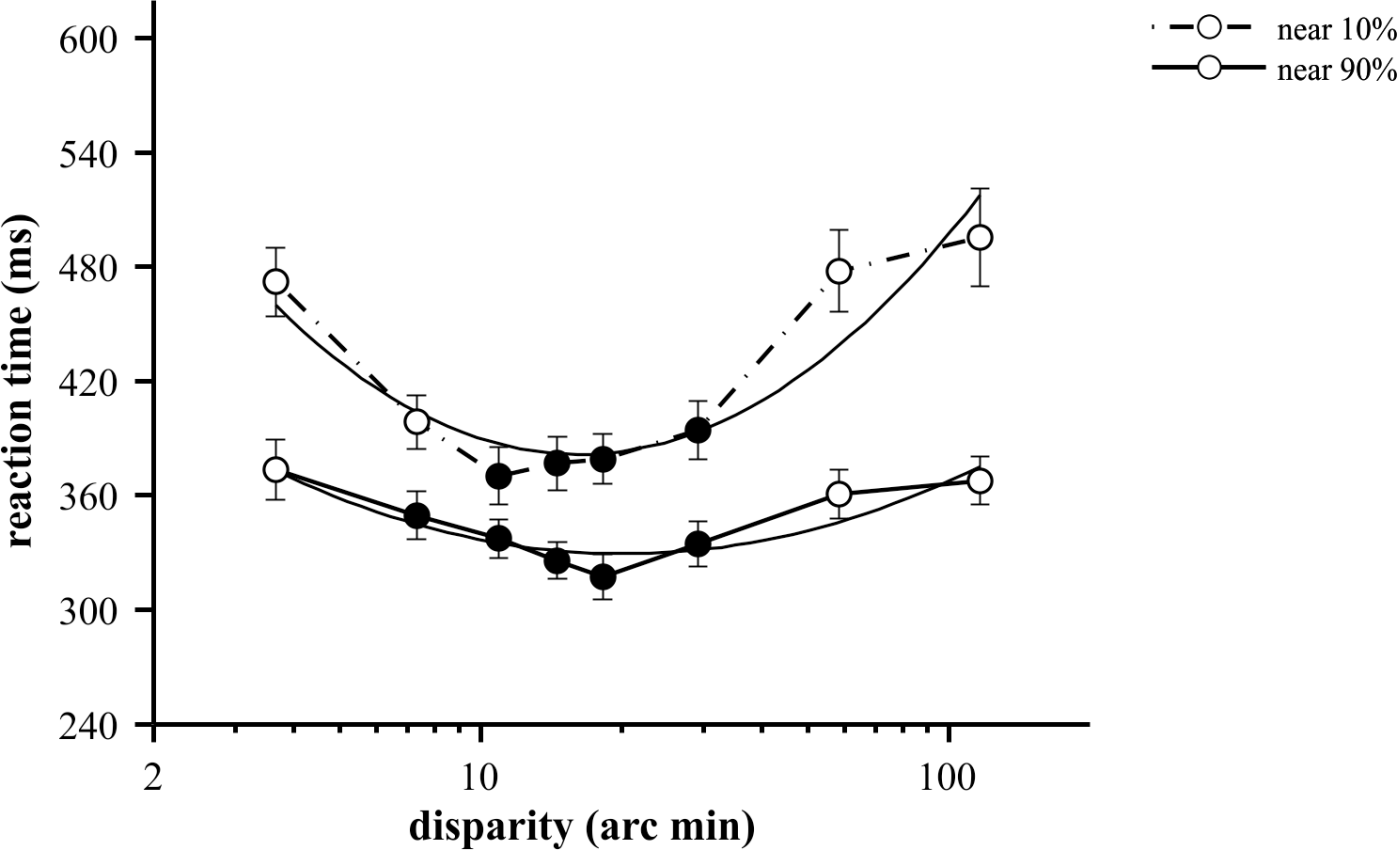
Mean reaction times for near disparity values at two (10% and 90%) contrast levels. Each data point represents the mean of 15 participants (at least 133 RTs); error bars show ±SEM. RTs formed statistically homogeneous groups for each contrast level. While the RTs (filled circles) were not significantly different from the shortest mean RT (370 ms for 10% and 317 ms for 90%), RTs signed open circles were not significantly different from the longest mean RTs (495 ms for 10% and 373 ms for 90% contrast), except 7.3 arc min at 10% contrast. Solid black curves show best fit 2^nd^ order polynomial functions (R^2^ = 0.867, min. value = 16.3 arc min, equation = 186 * *x*^2^ − 450 * *x* + 654 or 10% and R^2^ = 0.833, min. value = 20.2 arc min, equation = 79 * *x*^2^ − 206 * *x* + 464 for 90% contrast).

**Fig 3:**
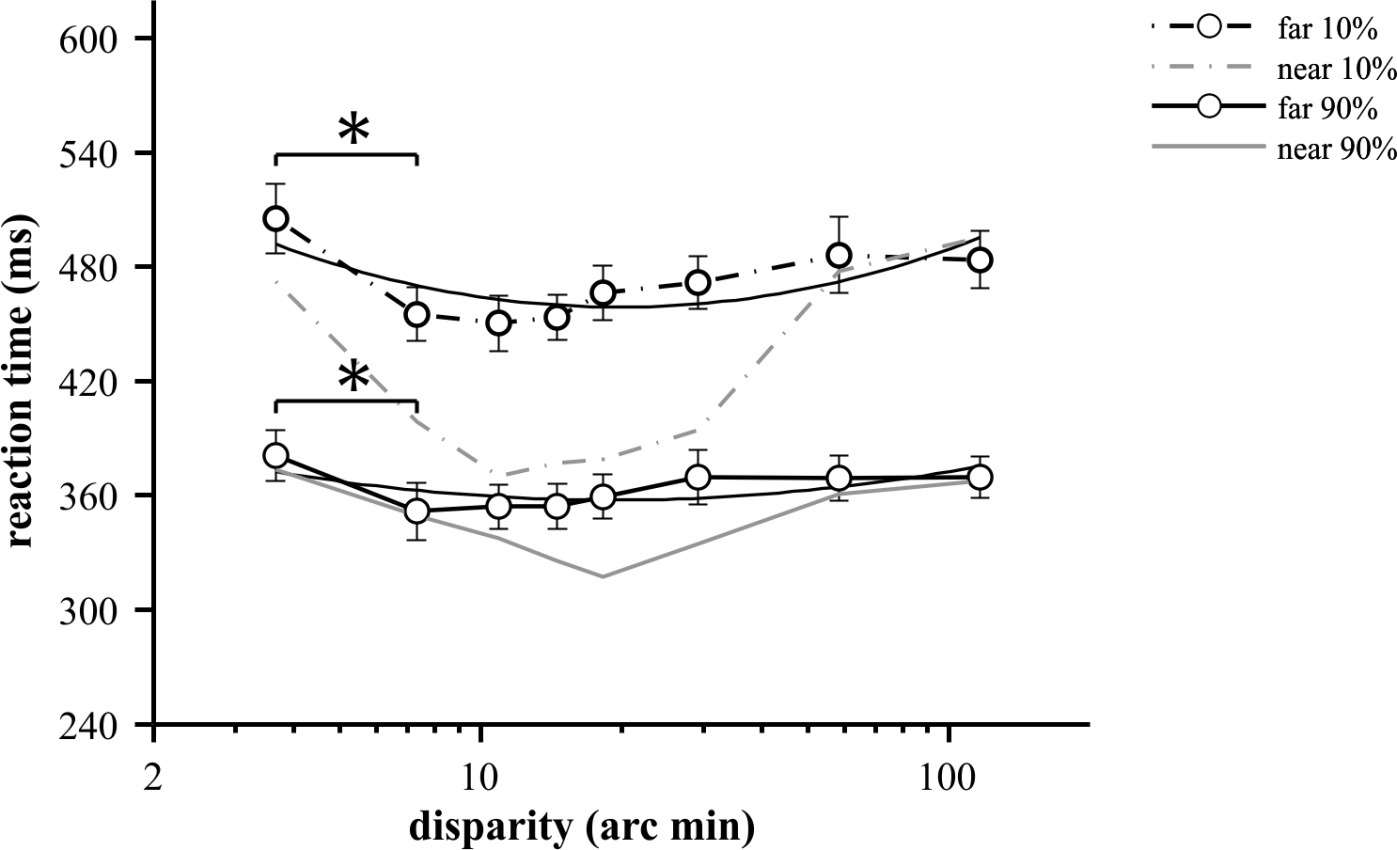
Mean reaction times for far disparity values at two contrast levels (10% and 90%). Each data point represents the mean of 15 participants (at least 141 RTs); error bars show ±SEM. Asterisk marks significant difference (p<0.05) found in pairwise comparisons between the lowest disparity and disparity with the shortest mean RT at both contrast. The mean RTs for near disparities (from Fig 2) are superimposed for comparison by gray lines. Second order polynomial fits are represented by solid black curves (R^2^ = 0.592, min. value = 19.8 arc min, equation = $61.3*x^{2}-159*x+562\;$for 10% and R^2^ = 0.422, min. value = 19 arc min, equation = $28.8*x^{2}-74*x+405\;$for 90% contrast).

**Fig 4:**
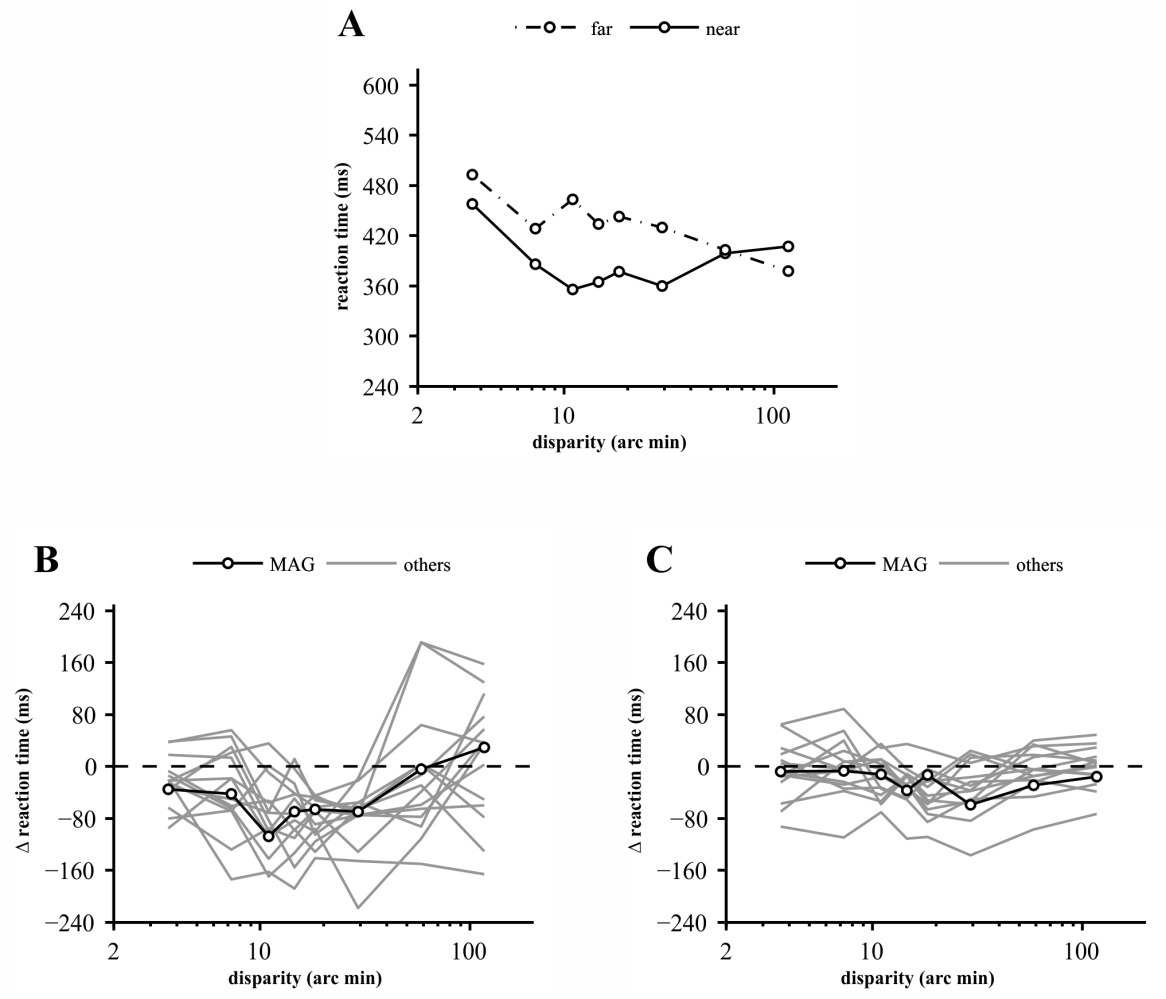
The difference between reaction times to near and far disparity DRDS checkerboards at 10% and at 90% contrast. **(A)** Median reaction times of participant MAG for near (solid lines) and far (broken lines) disparity DRDS checkerboards at 10% contrast. Each data point represents the median of up to 10 valid responses. This participant responded slower to far targets of all disparity magnitudes except for the largest one. **(B)** The difference between reaction times to near and far disparity DRDS checkerboards at 10% contrast for all participants. Data of participant MAG (shown in A) are plotted by solid black line and open circles; the remaining participants are plotted by gray lines. Note that reaction times to near disparities were always shorter in the middle of the tested disparity range. **(C)** The same as **B** for 90% contrast.

**Fig 5:**
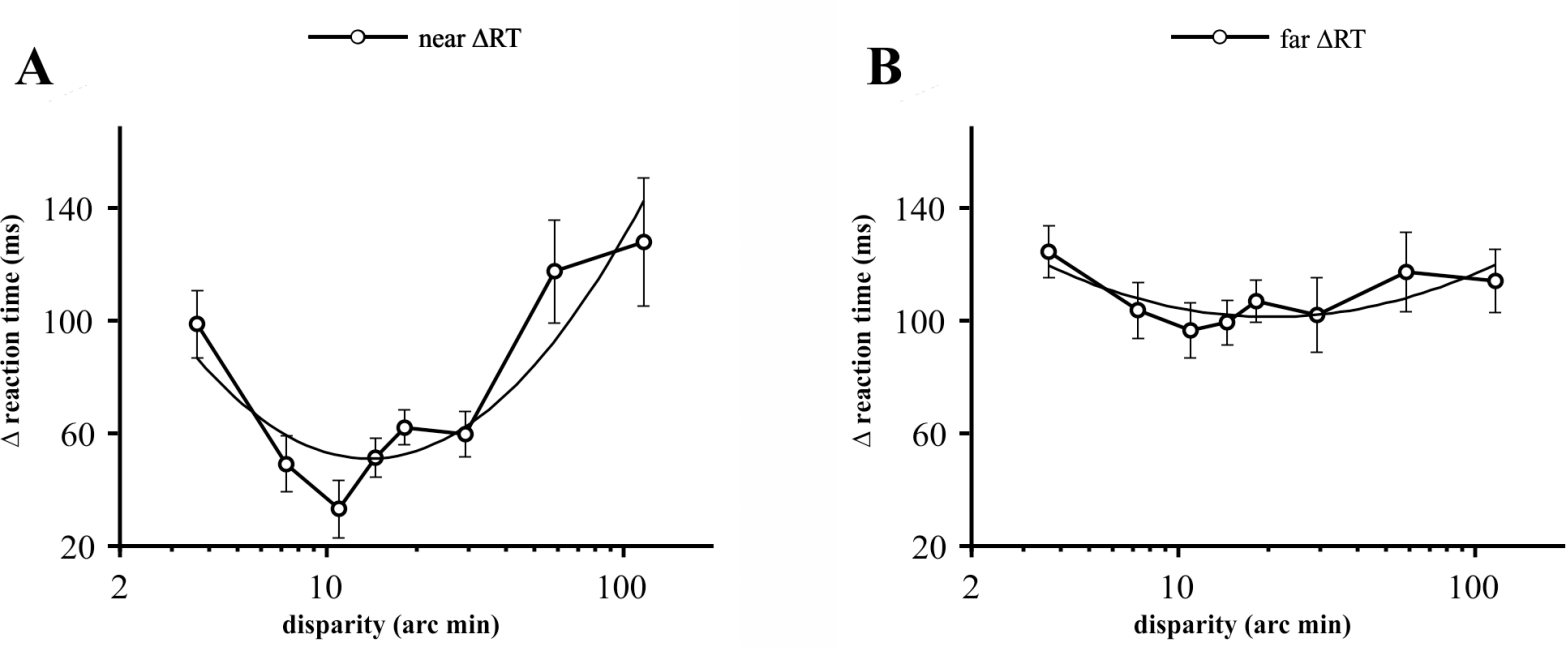
The difference between ΔRTs to near and far disparity. **(A)** Differences of mean RT values between 10% and 90% contrast (ΔRT) for near disparities. The data points represent means of 15 participants, error bars show ±SEM. The best fit 2^nd^ order polynomial function (solid black curve) is shown (R^2^ = 0.821). **(B)** The same as **A** for far disparities, (R^2^ = 0.62).

### Reaction times

The aim of these experiments was to characterize the effect of three variables on simple reaction times: disparity magnitude, the type of disparity, i.e. near or far and stimulus contrast. The rANOVA test of RTs with disparity and contrast as factors indicated significant main effects of both of these variables for near as well as far types of disparity (p<0.02, Figs 2 and 3, S1 Table). This statistical result could be explained on the one hand, by the characteristic U-shaped disparity tuning, and on the other hand by the reduction of RTs as a function of contrast. The disparity tuning of reaction times was most pronounced for near disparities at low (10%) contrast. Both far disparity and increased contrast reduced this effect, which is most evident in the almost flat tuning curve for far disparities at high contrast (Fig 3).

### Effect of disparity on reaction times

Here, we provide a detailed analysis of effects of disparity magnitude on reaction times separately for near and far disparities; relationships to stimulus contrast will be considered next.

At near disparities, participants responded with the shortest RTs to stimuli at 18 (317 ms) and 11 (370 ms) arc min disparity for 90% and 10% contrast level, respectively. The characteristic U-shaped disparity tuning of RT is represented on Fig 2. The figure shows averaged median RT values of the participants (n = 15) at two different contrast levels plotted as a function of disparity.

Two statistical findings support the effect of disparity. First, the main effect of stimulus disparity was highly significant (F(2.12, 29.68) = 27.99, p<10^−6^, r = 0.667, S1 Table) in rANOVA. We performed pairwise comparisons (with Bonferroni correction) to establish which disparities produce significantly different RTs from the shortest mean RT. As shown by filled circles and the rectangles in Fig 2, disparities from 11 to 29 arc min at both contrast levels tested (p>0.05) were statistically homogeneous, thus they formed an optimum range. RTs at lower and higher disparities were not significantly different from each other (except 3.7 versus 7.3 arc min at 10% contrast, p<10^−4^).

Second, the disparity vs. RT function had a strong significant quadratic trend for both contrast levels tested (F(1,14) = 105.9, p<10^−6^, r = 0.883 for 90% and F(1,14) = 70.8, p<10^−5^, r = 0.835 for 10%, S2 Table). Fitting a second-order polynomial (Fig 2) resulted in an estimated optimum disparity of 15 arc min for 10% contrast (and 18 arc min for 90%). Accordingly, lower and higher disparity values took longer time to respond to.

The disparity tuning of mean RTs for far disparities is shown in Fig 3. Similarly to near disparities, the magnitude of disparity had a significant influence as confirmed by rANOVA (F(2.66,37.3) = 6.56, p = 0.002, r = 0.319, S1 Table). The shortest mean RTs were 450 ms at 11 arc min disparity for 10% and 351 ms at 7.3 arc min disparity for 90% (Fig 3) but these values were statistically not different from RTs obtained at other disparities (p>0.05, pairwise comparisons after Bonferroni-correction) except for the single data point at 3.7 arc min disparity at both contrasts. Thus, a statistically segregated optimum range could not be found.

The U-shape of the curve is suggested by a statistically significant quadratic tendency, that was found both at 90% (F(1,14) = 10.01, p = 0.007, r = 0.417) and 10% (F(1,14) = 13.52, p = 0.002, r = 0.491, S2 Table) contrast. However, modulation of RT by far disparities was clearly milder than it was for near disparities. This is evidenced by the fact that the coefficient of the second order term in the fitted polynomial was 185.7 for near and 61.3 for far disparities at 10% contrast.

Individual data sets showed consistently longer RTs for far versus near disparities, especially in the medium range at 10% contrast. This is illustrated by the RTs of participant MAG (Fig 4A) who responded slower to all far targets except the highest one. (S)he was chosen as representative because (s)he had the least RMS deviation from the mean near vs. far RT difference (Δ reaction time, Fig 4B) of all participants. Fig 4B and 4C plot near–far RT differences for all participants at low and high contrasts, respectively. The data showed essentially the same tendency with two disparity magnitudes (18 and 29 arc min) producing slower (by 18–219 ms) far responses in all participants with a single exception at 90% contrast.

Mean RTs of all participants for near and far disparities are compared in Fig 3. The clearest differences are revealed at 10% contrast where the effect of near versus far disparities is the largest in the optimum range of the tuning curves. This observation is confirmed by rANOVA with the magnitude and type of disparity as factors. Both the type of disparity (p = 0.0003), the disparity magnitude (p<10^−6^, S4 Table) and the interaction of factors (p = 0.0008) had a strongly significant influence.

We performed paired t-tests on RTs at each disparity magnitude to highlight the range where near/far differences were significant (S5 Table). At 10% contrast, mean RT-pairs were different (p<0.05) from 3.7 to 29 arc min disparity whereas at 90% contrast, significant differences were found at 15 and 18 arc min disparities (p<0.05). Thus we believe that the actual effect of far disparity is a reduction of processing speed essentially to the level of non-fusible large disparities.

### Effect of contrast on reaction times

The main effect of contrast was significant for both types of disparities (rANOVA, F(1,14) = 100, p<10^−6^, r = 0.877 for near and F(1,14) = 279.2, p<10^−6^, r = 0.952 for far). The overall contrast effect can be easily observed on Figs 2 and 3: lower contrast values consistently resulted in longer RTs and less variance. Moreover, the disparity dependent change in RT was most pronounced at the lower (10%) contrast level, whereas at 90%, RT modulation by either disparity magnitude or near/far condition was strongly attenuated.

In order to quantify the effect of contrast, we first analyzed reaction time differences (ΔRT) between the two contrast levels. In a second step, we estimated the slope of the Piéron-function (Eq 1) describing RT vs. contrast relationship.

For near disparities, the contrast dependent mean RT differences were not uniform as a function of disparity, rather, again they had a strong significant quadratic tendency (F(1,14) = 24.9, p = 0.0002, r = 0.640, see Fig 5A, S3 Table).

At far disparities, mean RTs were shorter by about 96 to 124 ms when contrast was increased from 10% to 90% (Fig 5B). However, ΔRTs did not show any significant quadratic trend (S3 Table).

The increase in reaction time for extreme disparities or lower contrasts is a sign that stimuli became increasingly difficult to detect. Indeed, observers reported more difficulty especially in perceiving higher disparities, an observation that was confirmed by data analysis. Tables 1 and 2 show that the number of stimuli “not seen” by the participants in two consecutive trials (see Methods) was highest for the greatest disparities.

### Comparison of contrast gains

Simple reaction times to visual stimuli are known to depend on stimulus contrast according to a hyperbolic relationship described by Plainis and Murray [16] (Eq 1), a case of Piéron’s Law. As suggested by the same authors, an RT-based measure of contrast gain can be defined as the reciprocal of the slope k. This value can be interpreted in a similar way as contrast gains measured by neurophysiological methods [16, 17]: higher values represent higher increase in response for a unit change of contrast. Although we only tested two contrast levels in our experiment, the previous results inspired us to estimate RT-based contrast gains to illustrate contrast sensitivity differences between near and far disparities.

Thus, the contrast gain k^–1^ was calculated as

$$k^{-1}=\left(\frac{1}{C_{low}}-\frac{1}{C_{high}}\right)/\Delta RT$$ (2)

for each disparity, where ΔRT is the difference in RTs measured between the two contrasts and $\frac{1}{C_{low}}-\frac{1}{C_{high}}=\frac{1}{0.1}-\frac{1}{0.9}=0.89$ in all conditions. It can be seen from the formula that larger RT increments for a unit decrement of contrast result in higher slopes and lower gains.

**Fig 6:**
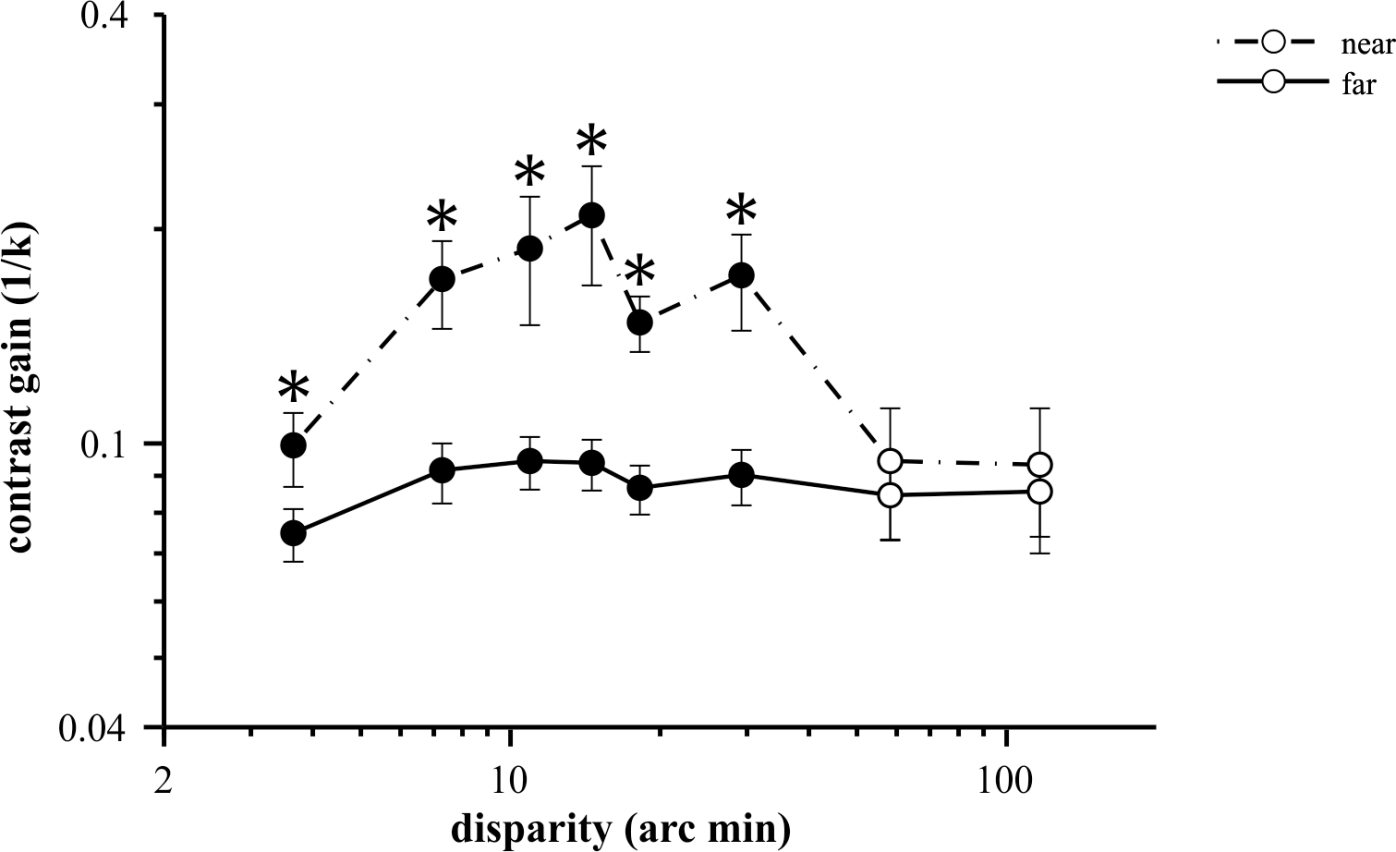
Representation of the RT-based contrast gain k^-1^ for near and far disparities. Solid line shows the near and the dashed line the far disparities. Asterisks mark disparity values where the contrast gains for near and far stimuli were significantly different (*p<0.05, paired t-test of log transformed data). The data points represent means of 15 participants, error bars show ±SEM.

Fig 6 plots RT-based contrast gains for near and far disparities in comparison. Contrast gain was the highest at 15 arc min disparity for near disparity. Statistical analysis confirmed that contrast gain was significantly different at disparities between 3.65 to 29 arc min (see asterisks, two-sample t-tests on log transformed data, p<0.043, S6 Table). This corresponded to the range around the estimated optimum for near disparities (Fig 2) and also where near/far RT differences were the greatest at 10% contrast (Fig 3). Taken together, the data suggest markedly different contrast sensitivity of processing near and far disparities.

## Discussion

To our knowledge, this is the first study examining the effect of disparity and contrast on simple RTs for dynamic random dot stereo-checkerboard patterns. The most important novel findings are the following: 1) Reaction times as a function of disparity show a strong U-shaped tuning with an optimum at around 15 arc min disparity; 2) contrast sensitivity for near disparities estimated as RT-based contrast gain follows the same U-shape course as RTs; 3) disparity tuning of RTs as well as contrast gain is asymmetric between near (crossed) and far (uncrossed) disparities.

By definition, reaction time (RT) is the time elapsed between the presentation of a stimulus and the subsequent behavioral response. Simple RTs, rather than more complex, task related choice or decision RTs, have two components: 1) early perceptual component corresponding to the detection of the target stimulus and 2) the motor response, e.g., the press of a button. Electrophysiological measurements of the response-locked lateralized readiness potential revealed that in simple RT tasks, the duration of the motor response can be regarded as more or less invariant in well cooperating participants. Thus, changes in RT of a given individual provide information about differences in the duration of early perceptual processing of the target stimuli [35]. In our experiments, participants were required to respond to the appearance of the target checkerboard but they did not have to discriminate its properties such as near or far nor had they to make cognitive decisions. Our data therefore characterize an early, low-level stage of stereoscopic processing.

The asymptote of the mean simple visual RTs to detect the onset of a change in luminance or contrast is typically around 210ms in young healthy individuals [36]. It has been shown that disparity signals are already available for perception between 100 and 200 ms after stimulus onset [37, 38]. On the other hand, evoked potentials for cyclopean stimuli show longer latencies compared to contrast defined patterns [39–44]. In addition, disparity sensitive neurons show about 25ms longer response latency to cyclopean stimuli in comparison with monocularly detectable visual cues in single unit studies [45]. Consistently, we found that even at the peak of the disparity tuning curve and at high contrast, mean RT was not less than 330ms.

In the following, we consider the sources of RT differences as a function of the three parameters that we manipulated: stimulus contrast, disparity magnitude and the type of disparity. It has been well known from the early work of Piéron that RTs are inversely proportional to stimulus intensity [46]. In line with earlier studies [16], we found a marked decrease in RT with increasing contrast (Figs 2 and 3). By calculating RT based contrast gain, we could factor out the intensity (i.e. contrast) effect. The real advantage of measuring RTs at multiple contrasts is that the steepness of the contrast-RT relationship can differentiate between processing mechanisms with different contrast sensitivities, as it has been shown before e.g. for targets processed predominantly by the parvocellular or magnocellular streams [17]. How can then we explain the effect of disparity magnitude and near-far asymmetry?

First, it is worth noting that our stimuli contained, by design (see Methods), uncorrelated areas of increasing proportion at increasing disparities. Half of the disparate area of the target pattern was binocularly uncorrelated at the second to the last disparity (i.e., 60 arc min), whereas no corresponding dots were present in the stimulus at the highest tested disparity of 120 arc min. Importantly, this effect was equally present in near and far disparity targets so that it cannot account for the observed near-far asymmetry of RTs or contrast gains. Nevertheless, it might have contributed to the increase in reaction times on the right-hand side of the disparity tuning curve. However, disparities in excess of 1 degree are not possible to fuse for most participants and therefore an RDS of such disparity would normally appear as uncorrelated in any case. This is clearly illustrated in Figs 3, 4 and 6 showing that the near and far disparity tuning curves overlap at 60 arc min and above.

The U-shaped tuning of the RT-disparity function is not unexpected in the light of past research. Many visual functions have an optimum range of parameters such as spatial or temporal frequency, to which the responding neuronal elements are the most sensitive. However, there are remarkably little data available in the literature on the disparity sensitivity of simple measures of psychophysical performance. One obvious limit to the detection of stereoscopic target stimuli is the disparity threshold, which is in the order of 1/3 minutes of arc depending on contrast [47] as well as whether near or far disparities are concerned [24]. Even though we did not have measurements below 3 minutes of arc, RTs clearly increased towards low disparities as expected when approaching threshold.

An increase in RT was also expected in the large disparity range where binocular fusion is limited by the diplopia threshold (also called Panum’s fusional area). This limit was estimated to vary between 2 and 20 arc min with higher values measured at higher visual field eccentricity [23]. As the size of our stimuli (16°x16°) extended well beyond the foveal-parafoveal visual field, we can safely estimate the fusion limit to be close to 20 arc min. Consequently, our measurements suggest that RT is increased and contrast gain is reduced (Figs 2, 3, 4A and 6) as disparity overrides Panum’s fusional area.

Despite methodological differences, our results are also in agreement with disparity tuning curves of the human stereoscopic system obtained by visual evoked potentials [48–50] or functional magnetic resonance imaging [51]. The peak of the neuronal response was found between 10 and 16 arc min in these studies and responses declined towards smaller as well as greater disparities, similar to our RT-based curves (optima around 15 arc min, Fig 2).

More surprising than the U-shaped disparity tuning is our result that the same relationship is almost completely missing for far disparities. Asymmetries between the processing of near and far disparities have been demonstrated by several authors but the underlying causes have been debated. A study by Larson [52] using clinical stereovision tests for instance concluded that sensitivity differences to near and far disparities can vary individually. Schumer and Julesz explained individual asymmetries in disparity thresholds at near and far disparities by idiosyncratic differences in fixation disparity on the order of several minutes of arc [53]. According to their interpretation, the asymmetries may reflect no more than a systematic misconvergence to either near or far direction. If misconvergence randomly occurred in the population, one would expect near-far asymmetry to vary among participants. However, all our participants showed faster RTs to some range of near disparities and all participants were faster between 18 and 29 arc min (Fig 4). It is very unlikely that our randomly selected population of 15 young, healthy individuals had by chance, misconvergence in the same direction.

Our data is more compatible with studies reporting systematic near-far asymmetries in various psychophysical measures of stereoscopic processing [24–29]. Neurophysiological correlates of near-far asymmetries were reported by Sahinoglu [54] who found that the amplitudes of evoked potentials in response to near and far DRDS stimuli were negatively correlated in the disparity range between 2 and 12 arc min. Even more relevant to our study is the finding that VEP latencies were smaller by up to about 50ms for near disparities suggesting faster or more sensitive processing. In another study using high-density EEG and MR-based identification of several visual cortical areas, Cottereau et al. [50] found, apart from a U-shaped disparity tuning, larger visual evoked potentials in response to changes from far to near disparities as compared to the reverse change in visual cortical areas V1 and V3A.

Reaction times have recently been interpreted in the framework of the so-called integration-to-bound theory [55]. The model assumes a decision variable intercalated between early sensory processing and the motor systems, which accumulates sensory evidence until a certain threshold is reached that triggers a motor response. A neurophysiological correlate of the decision variable has been identified in primate single units as well as in the human EEG, as a centro-parietal positivity [56–60]. A neural model of faster RTs to near than far disparity targets might thus assume higher degree of input convergence onto the neurons implementing the decision variable. Indeed, several studies have found larger numbers of near cells in monkey visual areas such as MT [61], V3 [62] or V4 [63] in comparison to far cells. Computational models of near-far asymmetry of VEP amplitudes based on cell numbers have also been suggested [50, 64]. The same model could be envisaged to explain increased contrast sensitivity since the integration of a larger number of inputs is expected to increase signal-to-noise ratio [65]. Other possibilities to accelerate reaction times are higher activity or faster signal propagation in the sensory input pathway. Increased spike rates certainly contribute to the reduction of RTs at higher contrast. On the other hand, the decreased latency of VEPs evoked by near disparity targets [54] suggests that their signals also propagate faster in the cortical network.

The cyclopean stimuli used in our present study were checkerboard objects popping out or dropping behind the monitor plane. Both stimuli generate fast apparent motions either toward or away from the observer. Although a checkerboard is an artificial pattern, the direction of the motion itself carries biological significance. Objects that are moving away or toward the organism have different biological meaning to a certain species and they can be linked to opposite pairs of motivated behavior (e.g., fight/flight, approaching/distancing, go/no-go, consume/avoid, attend/neglect). Based on the motivational theory, an alternative explanation for the significant difference between RTs for near and far disparities is that fast motion toward and away from the individual may have different biological relevance. A similar idea was phrased by several authors; objects in the background may be useful in computing body motion whereas objects in front of the observer’s fixation plane pose a threat of colliding with the observer [54, 64, 66].

## Supporting information

S1 Table The statistical models for the results. (DOCX)

S2 Table Results of repeated measures ANOVA on reaction times as a function of disparity at near and far disparities and two contrast levels. (DOCX)

S3 Table Results of repeated measures ANOVA on contrast dependent reaction time differences (between 10% and 90% contrast) as a function of disparity at near and far disparities. (DOCX)

S4 Table Results of repeated measures ANOVA on reaction time differences between near and far targets as a function of disparity at 10% and 90% contrast. (DOCX)

S5 Table Results of paired samples t-test of reaction times to near vs. far stimuli at each of the tested disparity magnitudes and both tested contrast levels. (df = 14).
(DOCX)

S6 Table Results of paired samples t-test on log(contrast gain) measured for near vs. far stimuli at each of the tested disparity magnitudes. (df = 14).
(DOCX)

S7 Table The distribution of conditions where two repetitions were needed. (DOCX)

S8 Table Results of 3-factor (type of disparity, contrast, disparity magnitude) repeated measures ANOVA of our data set. (DOCX)

S1 Appendix Statistical coherence analysis. Appendix contains the description of the statistical coherence analysis based on the bootstrapping method to test (1) the number of RT measurements and (2) the number of participants needed to achieve statistical significance in the tests performed.
(PDF)

S1 Dataset Raw reaction times. The data set contains the 10 raw reaction times for each subject and condition in tab delimited text format. The very first raw of the file contains the field information. **ParticipantID:** anonymous participant ID (P01-P15); **Stimulus('):** disparity in arc min; **Contrast(%):** stimulus contrast was either 10 or 90%; **Reaction-Time:** reaction times were measured in ms; **Visibility:** “Seen” the reaction time was valid, “NotSeen” the reaction time was not available; **Disparity:** type of disparity “far” (i.e., uncrossed) or “near” (i.e., crossed) disparity.
(TXT)
