# Correction: Simple reaction times to cyclopean stimuli reveal that the binocular system is tuned to react faster to near than to far objects

**DOI:** 10.1371/journal.pone.0202358

**Published:** 2018-08-09

**Authors:** Gábor Horváth, Vanda A. Nemes, János Radó, András Czigler, Béla Török, Péter Buzás, Gábor Jandó

In the Introduction section, there is an error in the first equation. The variable *k* does not appear. Please view the complete, correct equation here:

$$RT=RT_{0}+k\frac{1}{C}$$

## References

[1] Horváth G, Nemes VA, Radó J, Czigler A, Török B, Buzás P, et al (2018) Simple reaction times to cyclopean stimuli reveal that the binocular system is tuned to react faster to near than to far objects. PLoS ONE 13(1): e0188895 10.1371/journal.pone.0188895 29304135 PMC5755738

