# Supplementary material for: Simple reaction times to cyclopean stimuli reveal that the binocular system is tuned to react faster to near than to far objects
Source: PLoS One. 2018 Jan 5;13(1):e0188895. doi: 10.1371/journal.pone.0188895 (PMC5755738; doi:10.1371/journal.pone.0188895)
Supplement: S1 Table — (DOCX) [file pone.0188895.s001.docx]

| **result section** | **statistical model** |
| --- | --- |
| **Reaction times** |  |
|  | RTs tested with 2x8 rANOVA (contrast*disparity) for near |
|  | RTs tested with 2x8 rANOVA (contrast*disparity) for far |
| **Effect of disparity on reaction times** |  |
| Near disparities |  |
|  | RTs tested with 2x8 rANOVA (contrast*disparity) for near |
|  | RTs tested with 1x8 rANOVA (disparity) for near, 90% contrast (S2 Table) |
|  | *- pairwise comparison with Bonferroni correction* |
|  | *- quadratic trend analysis (S2 Table)* |
|  | RTs tested with 1x8 rANOVA (disparity) for near, 10% contrast (S2 Table) |
|  | *- pairwise comparison with Bonferroni correction* |
|  | *- quadratic trend analysis (S2 Table)* |
| Far disparities |  |
|  | RTs tested with 2x8 rANOVA (contrast*disparity) for far |
|  | RTs tested with 1x8 rANOVA (disparity) for far, 90% contrast (S2 Table) |
|  | *- pairwise comparison with Bonferroni correction* |
|  | *- quadratic trend analysis (S2 Table)* |
|  | RTs tested with 1x8 rANOVA (disparity) for far, 10% contrast (S2 Table) |
|  | *- pairwise comparison with Bonferroni correction* |
|  | *- quadratic trend analysis (S2 Table)* |
| Comparison of near and far disparities |  |
|  | RTs tested with 2x8 rANOVA (type of disparity(near/far)*disparity) at 10% |
|  | contrast (S4 Table) |
|  | Paired t-test (near vs. far) at 90% and 10% contrast |
|  | (S5 Table) |
| **Effect of contrast on reaction times** |  |
|  | RTs tested with 2x8 rANOVA (contrast*disparity) for near |
|  | RTs tested with 2x8 rANOVA (contrast*disparity) for far |
|  | ΔRTs tested with 1x8 rANOVA (disparity) for near (S3 Table) |
|  | *- quadratic trend analysis (S3 Table)* |
|  | ΔRTs tested with 1x8 rANOVA (disparity) for far (S3 Table) |
|  | *- quadratic trend analysis (S3 Table)* |
| Comparison of contrast gains |  |
|  | log(gain) tested with two-sample t-test (S6 Table) |
