# Supplementary material for: Simple reaction times to cyclopean stimuli reveal that the binocular system is tuned to react faster to near than to far objects
Source: PLoS One. 2018 Jan 5;13(1):e0188895. doi: 10.1371/journal.pone.0188895 (PMC5755738; doi:10.1371/journal.pone.0188895)
Supplement: S2 Table — (DOCX) [file pone.0188895.s002.docx]

|  |  | **90% contrast** | | | | **10% contrast** | | | |
| --- | --- | --- | --- | --- | --- | --- | --- | --- | --- |
| **stim. type** | **statistic type** | **df (error)** | **F** | **p** | **r** | **df (error)** | **F** | **p** | **r** |
| near | rANOVA | 7 | 13.823 | <0.0001 | 0.497 | 2.135 | 21.903 | <0.0001 | 0.610 |
|  | (disparity) | (98) |  |  |  | (29.894) |  |  |  |
| near | rANOVA | 1 | 105.949 | <0.0001 | 0.883 | 1 | 70.757 | <0.0001 | 0.835 |
|  | (quadratic trend) | (14) |  |  |  | (14) |  |  |  |
| far | rANOVA | 3.452 | 3.790 | 0.012 | 0.213 | 3.352 | 4.745 | 0.004 | 0.253 |
|  | (disparity) | (48.327) |  |  |  | (49.724) |  |  |  |
| far | rANOVA | 1 | 10.013 | 0.007 | 0.417 | 1 | 13.523 | 0.002 | 0.491 |
|  | (quadratic trend) | (14) |  |  |  | (14) |  |  |  |
