# Supplementary material for: Simple reaction times to cyclopean stimuli reveal that the binocular system is tuned to react faster to near than to far objects
Source: PLoS One. 2018 Jan 5;13(1):e0188895. doi: 10.1371/journal.pone.0188895 (PMC5755738; doi:10.1371/journal.pone.0188895)
Supplement: S3 Table — (DOCX) [file pone.0188895.s003.docx]

| **stim. type** | **statistic type** | **df (error)** | **F** | **p** | **r** |
| --- | --- | --- | --- | --- | --- |
| near | rANOVA | 2.818 | 9.584 | <0.0001 | 0.406 |
|  | (disparity) | (39.449) |  |  |  |
| near | rANOVA | 1 | 24.857 | 0.0002 | 0.640 |
|  | (quadratic trend) | (14) |  |  |  |
| far | rANOVA | 7 | 1.158 | 0.334 | 0.076 |
|  | (disparity) | (98) |  |  |  |
| far | rANOVA | 1 | 3.679 | 0.076 | 0.208 |
|  | (quadratic trend) | (14) |  |  |  |
