# Supplementary material for: Simple reaction times to cyclopean stimuli reveal that the binocular system is tuned to react faster to near than to far objects
Source: PLoS One. 2018 Jan 5;13(1):e0188895. doi: 10.1371/journal.pone.0188895 (PMC5755738; doi:10.1371/journal.pone.0188895)
Supplement: S4 Table — (DOCX) [file pone.0188895.s004.docx]

| **contrast (%)** | **statistic type** | **df (error)** | **F** | **p** | **r** |
| --- | --- | --- | --- | --- | --- |
| 90 | rANOVA | 1 | 5.766 | 0.031 | 0.292 |
|  | (stim.type) | (14) |  |  |  |
| 90 | rANOVA | 2.817 | 10.933 | <0.0001 | 0.439 |
|  | (disparity) | (39.445) |  |  |  |
| 10 | rANOVA | 1 | 22.925 | 0.0003 | 0.621 |
|  | (stim.type) | (14) |  |  |  |
| 10 | rANOVA | 2.359 | 23.470 | <0.0001 | 0.626 |
|  | (disparity) | (33.025) |  |  |  |
