# Supplementary material for: Simple reaction times to cyclopean stimuli reveal that the binocular system is tuned to react faster to near than to far objects
Source: PLoS One. 2018 Jan 5;13(1):e0188895. doi: 10.1371/journal.pone.0188895 (PMC5755738; doi:10.1371/journal.pone.0188895)
Supplement: S5 Table — (df = 14). (DOCX) [file pone.0188895.s005.docx]

| **disparity (arc min)** | **contrast (%)** | **mean** | **std. dev.** | **t** | **p** |
| --- | --- | --- | --- | --- | --- |
| **3.7** | 10 | -33.267 | 57.230 | -2.251 | 0.041 |
| **7.3** | 10 | -56.567 | 54.226 | -4.040 | 0.001 |
| **11** | 10 | -80.200 | 68.911 | -4.507 | 0.000 |
| **15** | 10 | -76.467 | 39.345 | -7.527 | 0.000 |
| **18** | 10 | -87.133 | 33.165 | -10.175 | 0.000 |
| **29** | 10 | -77.400 | 52.720 | -5.686 | 0.000 |
| **58** | 10 | -8.200 | 103.081 | -0.308 | 0.763 |
| **120** | 10 | 11.867 | 86.310 | 0.532 | 0.603 |
| **3.7** | 90 | -7.533 | 43.706 | -0.668 | 0.515 |
| **7.3** | 90 | -2.067 | 45.690 | -0.175 | 0.863 |
| **11** | 90 | -16.833 | 31.840 | -2.048 | 0.060 |
| **15** | 90 | -28.367 | 30.928 | -3.552 | 0.003 |
| **18** | 90 | -42.200 | 34.701 | -4.710 | 0.000 |
| **29** | 90 | -35.000 | 42.480 | -3.191 | 0.007 |
| **58** | 90 | -8.500 | 34.974 | -0.941 | 0.363 |
| **120** | 90 | -1.967 | 30.555 | -0.249 | 0.807 |
