# Supplementary material for: Simple reaction times to cyclopean stimuli reveal that the binocular system is tuned to react faster to near than to far objects
Source: PLoS One. 2018 Jan 5;13(1):e0188895. doi: 10.1371/journal.pone.0188895 (PMC5755738; doi:10.1371/journal.pone.0188895)
Supplement: S6 Table — (df = 14). (DOCX) [file pone.0188895.s006.docx]

| **disparity (arc min)** | **mean** | **std. dev.** | **t** | **p** |
| --- | --- | --- | --- | --- |
| **3.7** | 0.125 | 0.216 | 2.230 | 0.043 |
| **7.3** | 0.271 | 0.267 | 3.930 | 0.002 |
| **11** | 0.306 | 0.380 | 3.119 | 0.008 |
| **15** | 0.334 | 0.291 | 4.452 | 0.001 |
| **18** | 0.239 | 0.152 | 6.084 | <0.001 |
| **29** | 0.265 | 0.359 | 2.859 | 0.013 |
| **58** | 0.045 | 0.377 | 0.466 | 0.649 |
| **120** | 0.038 | 0.381 | 0.386 | 0.705 |
