# Supplementary material for: Simple reaction times to cyclopean stimuli reveal that the binocular system is tuned to react faster to near than to far objects
Source: PLoS One. 2018 Jan 5;13(1):e0188895. doi: 10.1371/journal.pone.0188895 (PMC5755738; doi:10.1371/journal.pone.0188895)
Supplement: S7 Table — (DOCX) [file pone.0188895.s007.docx]

|  | **90% contrast level** | | | | | | | | **10% contrast level** | | | | | | | |
| --- | --- | --- | --- | --- | --- | --- | --- | --- | --- | --- | --- | --- | --- | --- | --- | --- |
| **disparity (arc min)** | 3.7 | 7.3 | 11 | 15 | 18 | 29 | 58 | 120 | 3.7 | 7.3 | 11 | 15 | 18 | 29 | 58 | 120 |
| **near** | 4 | 2 | 2 | 4 | 2 | 1 | 3 | 1 | 19 | 9 | 2 | 4 | 6 | 10 | 24 | 30 |
| **far** | 4 | 1 | 2 | 2 | 0 | 3 | 4 | 3 | 21 | 9 | 13 | 7 | 8 | 9 | 22 | 24 |
| **total** | 8 | 3 | 4 | 6 | 2 | 4 | 7 | 4 | 40 | 18 | 15 | 11 | 14 | 19 | 46 | 54 |
