# Supplementary material for: Simple reaction times to cyclopean stimuli reveal that the binocular system is tuned to react faster to near than to far objects
Source: PLoS One. 2018 Jan 5;13(1):e0188895. doi: 10.1371/journal.pone.0188895 (PMC5755738; doi:10.1371/journal.pone.0188895)
Supplement: S8 Table — (DOCX) [file pone.0188895.s008.docx]

| **statistic type** | **df (error)** | **F** | **p** | **r** |
| --- | --- | --- | --- | --- |
| rANOVA | 1 | 15.590 | 0.001 | 0.527 |
| (type of disp.) | (14) |  |  |  |
| rANOVA | 1 | 205.901 | <.0001 | 0.936 |
| (contrast) | (14) |  |  |  |
| rANOVA | 2.258 | 23.749 | <.0001 | 0.629 |
| (disp. value) | (31.618) |  |  |  |
| rANOVA | 1 | 33.001 | <.0001 | 0.702 |
| (type of disp.*contrast) | (14) |  |  |  |
| rANOVA | 2.402 | 10.828 | 0.0001 | 0.436 |
| (type of disp.*disp. value) | (33.625) |  |  |  |
| rANOVA | 7 | 11.582 | <.0001 | 0.453 |
| (contrast*disp. value) | (98) |  |  |  |
| rANOVA | 4436.058 | 2.923 | 0.024 | 0.173 |
| (type of disp.*contrast*disp. value) | (1515.511) |  |  |  |
| QUADRATIC TREND | 1 | 77.539 | <.0001 | 0.847 |
| (disp. value) | (14) |  |  |  |
| QUADRATIC TREND | 1 | 65.059 | <.0001 | 0.823 |
| (type of disp.*disp. value) | (14) |  |  |  |
| QUADRATIC TREND | 1 | 26.826 | 0.0001 | 0.657 |
| (contrast*disp. value) | (14) |  |  |  |
| QUADRATIC TREND | 1 | 7.842 | 0.014 | 0.359 |
| (type of disp.*contrast*disp. value) | (14) |  |  |  |
