# Supplementary material for: Simple reaction times to cyclopean stimuli reveal that the binocular system is tuned to react faster to near than to far objects
Source: PLoS One. 2018 Jan 5;13(1):e0188895. doi: 10.1371/journal.pone.0188895 (PMC5755738; doi:10.1371/journal.pone.0188895)
Supplement: S1 Appendix — Appendix contains the description of the statistical coherence analysis based on the bootstrapping method to test (1) the number of RT measurements and (2) the number of participants needed to achieve statistical significance in the tests performed. (PDF) [file pone.0188895.s009.pdf]

Statistical coherence analysis

Here, we describe a comprehensive statistical coherence analysis (SCA) based on the bootstrapping method to test (1) the number of RT measurements and (2) the number of participants needed to achieve statistical significance in the tests performed. We prove that 15 participants and 10 RTs per participant and condition were sufficient to achieve significant statistical outcome and that higher number of subjects in the population can compensate for the relatively few number of trials per subject. Furthermore, we prove that results obtained with any of the three measures of central tendency (median, arithmetic mean and harmonic mean) run very close to each other. This suggests that the individual distributions were close to Gaussian and especially they were not skewed to a degree that would be relevant for the final conclusions of the paper.

Theoretical considerations of SCA

We reasoned that if 10 RT measurements per condition were insufficient, then randomly drawing less than 10 RT's from our database and performing the same statistical procedures on them would quickly deteriorate p-values and reach values above the chosen threshold value ($p=0.05$). If on the other hand, 10 RT's are on the safe side, then p-values should converge to an acceptable level (e.g. $p<0.05$) already before reaching 10 RT's per condition. This idea is illustrated in S1 Fig. We can test the necessary number of participants in a similar way.

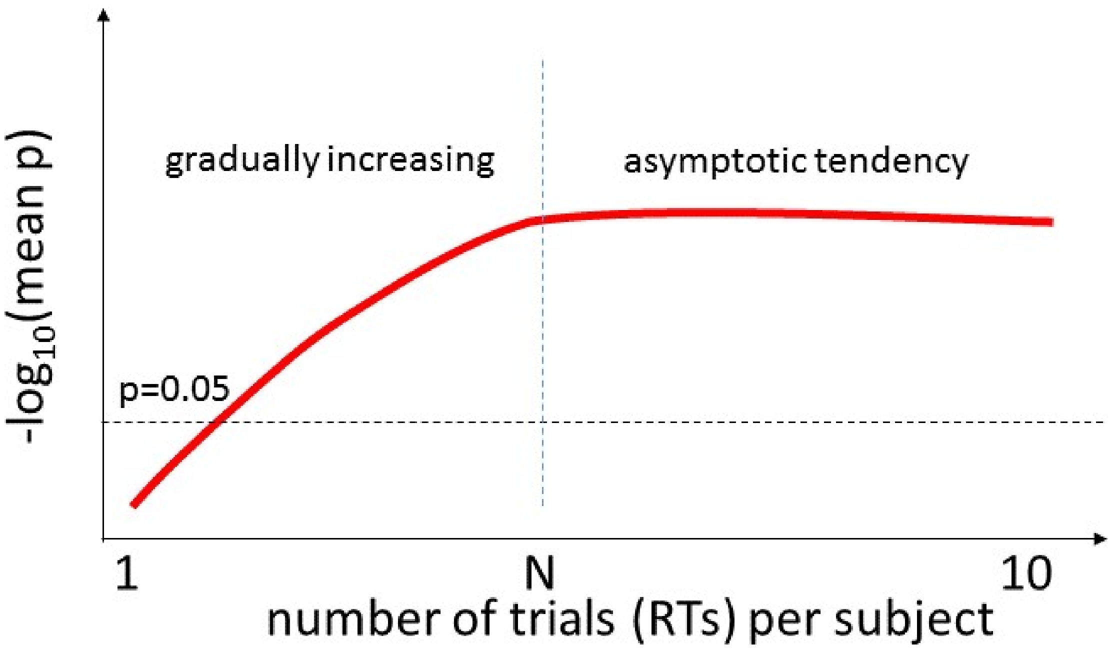

S1 Fig. Theoretical concept of the SCA. We test the hypothesis that less than N<10 RT measurements per condition and participant are sufficient to reach an asymptotic level of p-values. If this is true, a further increase in the number of RT measurements is not warranted by the data. We shall report the negative logarithm of the resulting p-values in order to cover several orders of magnitude on the vertical axis.

Method of SCA

Based on the above theoretical consideration, we systematically varied the number of trials per subject (i.e., $N_T \in \{1 \dots 10\}$) as well as the number of subjects (i.e., $N_S \in \{2 \dots 15\}$). This was done by drawing N_S subjects and N_T of their RT's randomly from our dataset 500 times for each combination of N_S and N_T. This resulted in $14 * 10 * 500 = 70000$ random subsamples. We then performed all seven ANOVA's reported in the paper on these datasets.

Now, we can answer the question what could have been the outcome of the experiment if we had performed the experiment with less than 15 subjects and less than 10 trials per subjects. We can also predict the variance of the mean theoretical statistical outcome, since each subsample was generated 500 times.

In addition, we could address another potential concern, namely whether the median is a good measure of each individual's RT. We therefore performed these calculations not only for the median but also for two other measures of central tendency, the arithmetic mean and the harmonic mean. The entire analysis thus involved running $70000 * 3 * 7 = 1470000$ ANOVA's.

Results of the SCA

S2-S8 Figs show the results of this analysis for the repeated measures ANOVAs reported in the Results section of our paper. We report the negative logarithm of p-values in order to cover several orders of magnitude on the vertical axis. On each graph, we plotted the average –log p-values of the 500 different random subsamples together with their SD's indicated by the error bars. The significance level of p=0.05 is shown by horizontal broken lines. The panels represent increasing number of participants (N_S) from top left ($N_S = 2$) to bottom right ($N_S = 15$). The horizontal axis of each panel represents the number of trials (N_T) per subject and condition.

Three findings are apparent from the figures. First, increasing the number of participants resulted in decreasing p-values (increasing -log p). Second, increasing the number of RTs taken for each condition and subject resulted in an asymptotic tendency in p-values. Interestingly, the statistical confidence does not seem to improve above 5-8 trials per subject and condition. The results of all 7 ANOVA's were similar in that statistical significance was reached below (or often well below) 10 RT's per subject for 15 participants.

The third outcome of the SCA was that results obtained with the three measures of central tendency (median, arithmetic and harmonic mean) run very close to each other.

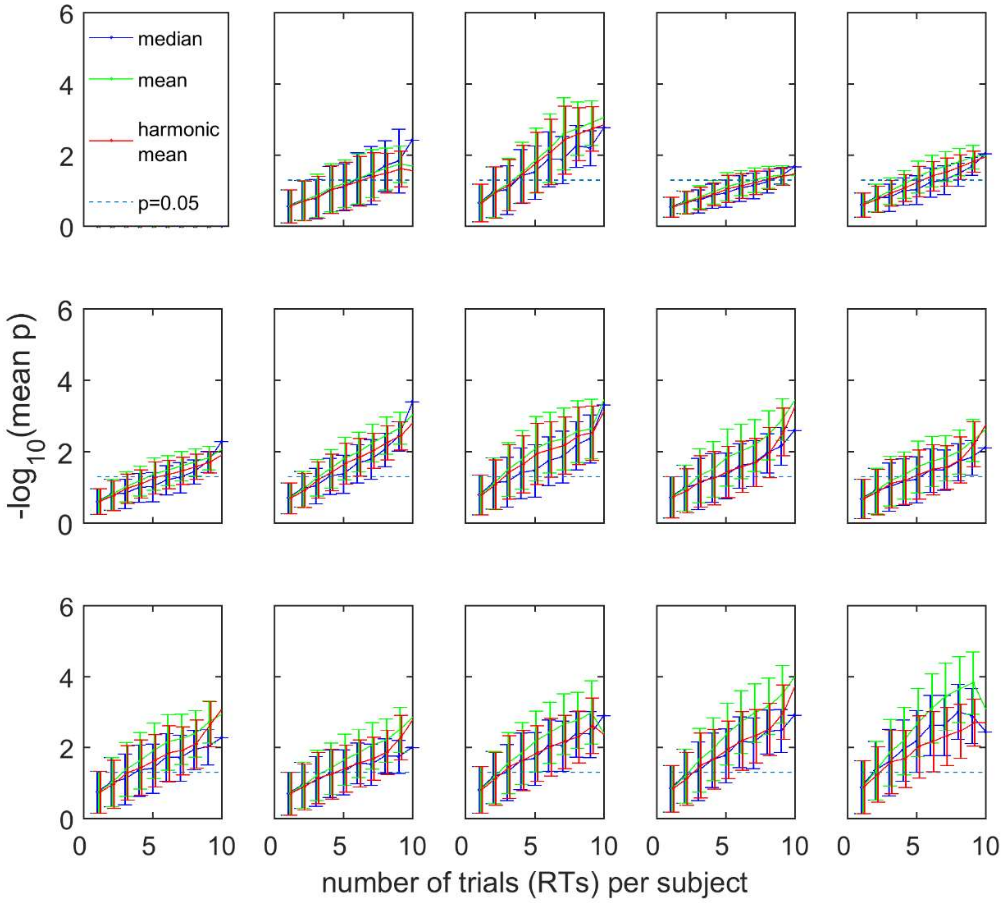

S2 Fig. SCA of the disparity main effect for far disparities (at 10% contrast). On each panel, we plotted the average –log p-values of the 500 different random subsamples against the number of trials (N_T) per subject and condition. Error bars indicate SD. The significance level of p=0.05 is shown by horizontal broken lines. Blue, green and red curves show results if the N_T reaction time measurements were represented in the further analysis by their median, arithmetic or harmonic mean, respectively. The panels represent increasing number of participants (N_S) from top left ($N_S = 2$) to bottom right ($N_S = 15$).

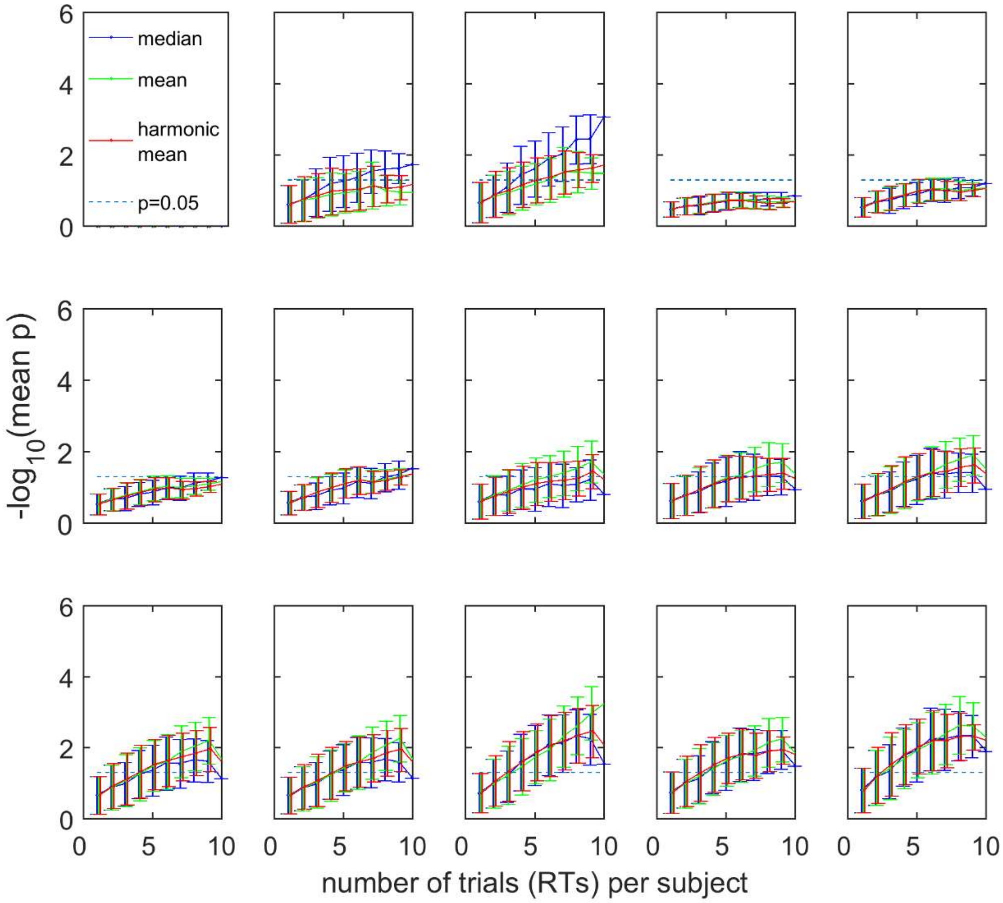

S3 Fig. SCA of the disparity main effect for far disparities (at 90% contrast). For further details, see S2 Fig.

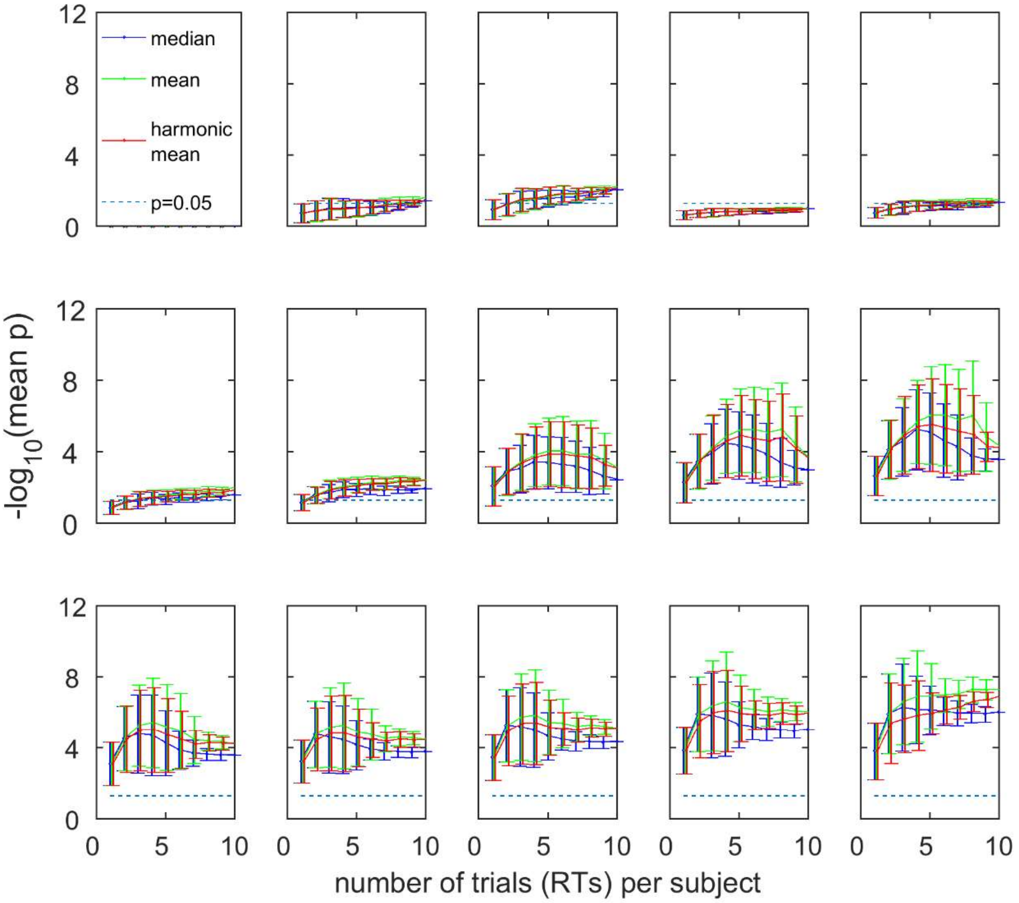

S4 Fig. SCA of the disparity main effect for near disparities (at 10% contrast). For further details, see S2 Fig.

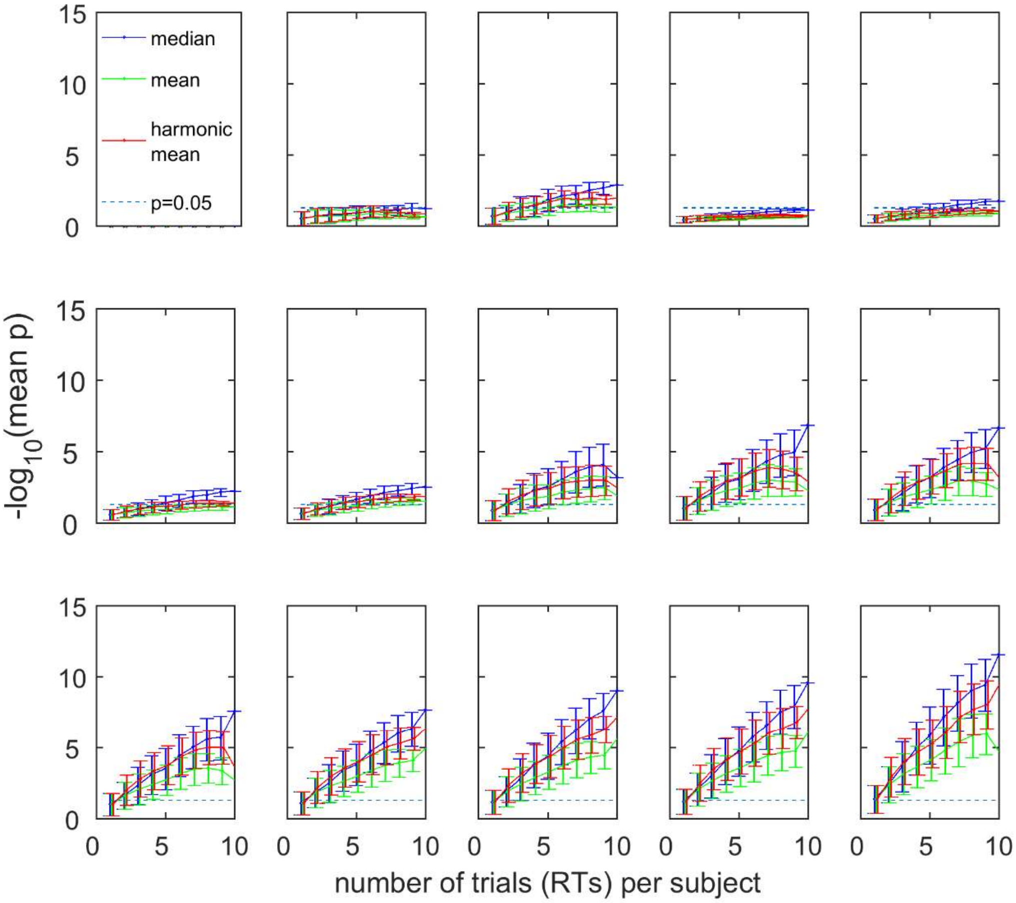

S5 Fig. SCA of the disparity main effect for near disparities (at 90% contrast). For further details, see S2 Fig.

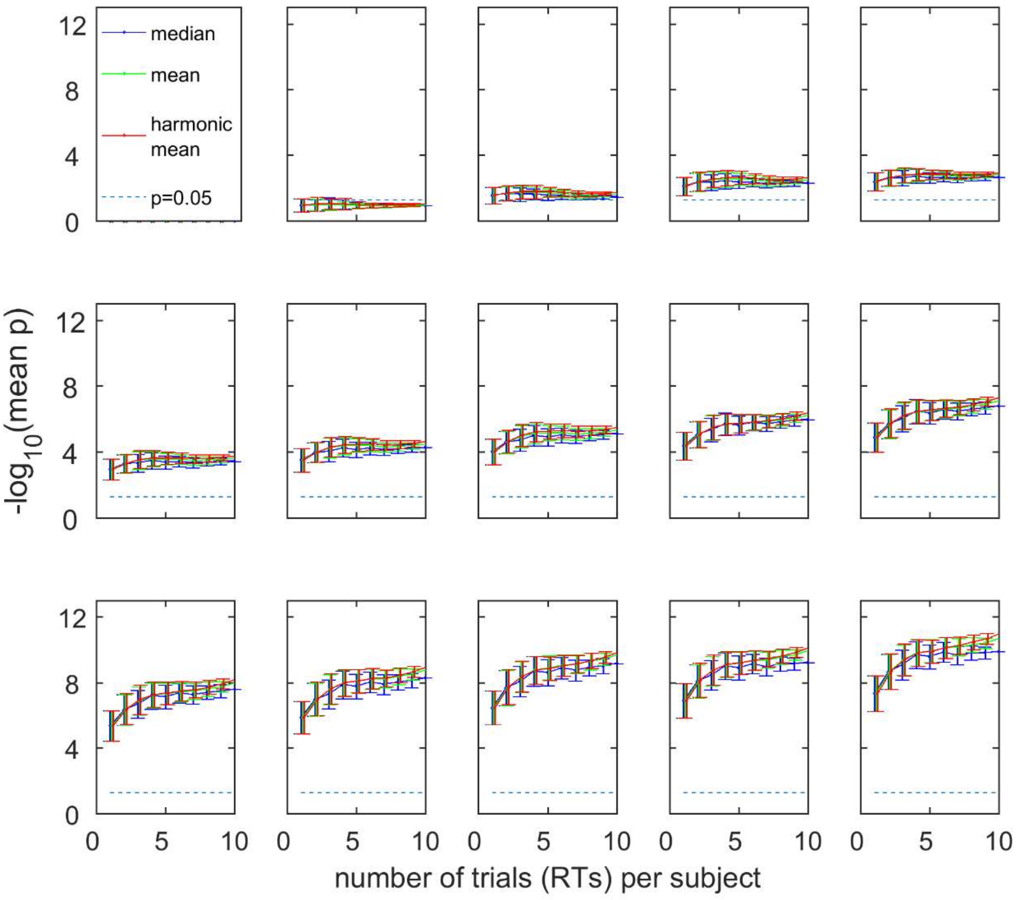

S6 Fig. SCA of the contrast effect for far and near disparities (10% vs. 90%). For further details, see S2 Fig.

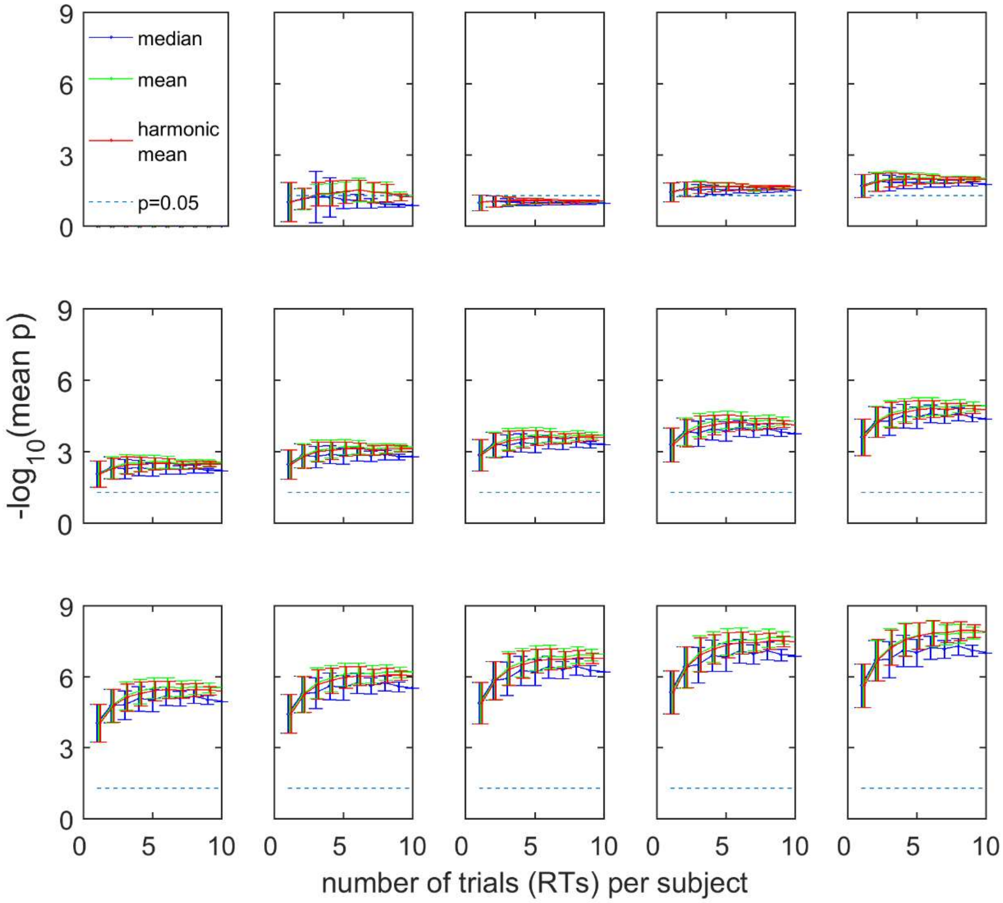

S7 Fig. SCA of the contrast effect for near disparities. (10% vs. 90% contrast). For further details, see S2 Fig.

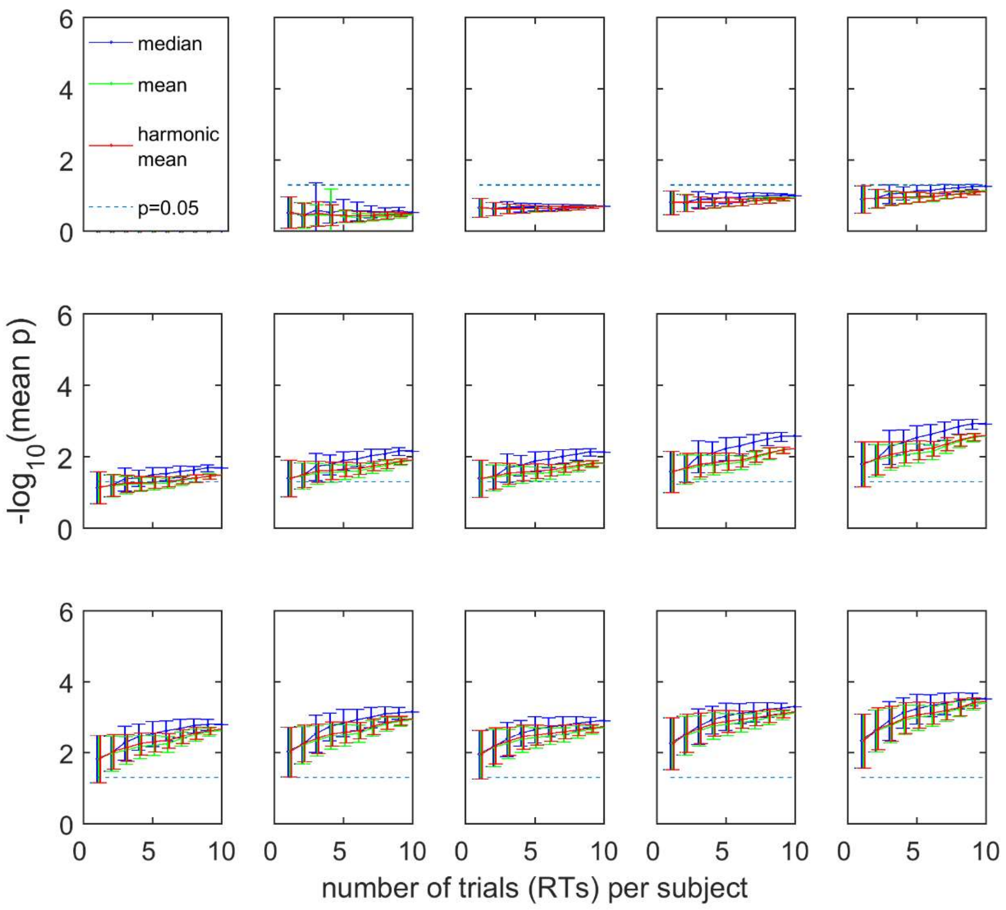

S8 Fig. SCA of the type of disparity effect at 10% contrast. (near vs. far). For further details, see S2 Fig.

Conclusion of SCA

The fact that using arithmetic mean or median as a measure of central tendency for individual RTs delivered very similar results demonstrates that the individual RT distributions were not skewed to a degree that would influence ANOVA results. This finding essentially confirms the use of medians (although means would have worked equally well).

Our analysis also demonstrates that increasing the number of participants can compensate for a lower number of trials and vice versa. It is clear from the graphs that we were on the safe side with respect to both parameters. In a more general sense, we have proved that it does not matter for the statistical model if we measure few or even just one RT from each individual and condition but we obtain data from a sufficiently large population or we measure many RTs from fewer individuals. The analysis has demonstrated that p-values reach a plateau at even less than 10 RT measurements per individual and condition. It can thus be predicted that increasing the number of trials would not improve statistical confidence.
